# Delivery of Insulin via Skin Route for the Management of Diabetes Mellitus: Approaches for Breaching the Obstacles

**DOI:** 10.3390/pharmaceutics13010100

**Published:** 2021-01-14

**Authors:** Abdul Ahad, Mohammad Raish, Yousef A. Bin Jardan, Abdullah M. Al-Mohizea, Fahad I. Al-Jenoobi

**Affiliations:** Department of Pharmaceutics, College of Pharmacy, King Saud University, P.O. Box 2457, Riyadh 11451, Saudi Arabia; mraish@ksu.edu.sa (M.R.); ybinjardan@ksu.edu.sa (Y.A.B.J.); amohizea@ksu.edu.sa (A.M.A.-M.); aljenobi@ksu.edu.sa (F.I.A.-J.)

**Keywords:** chronic disease, diabetes, insulin, skin, stratum corneum, transdermal

## Abstract

Insulin is used for the treatment of diabetes mellitus, which is characterized by hyperglycemia. Subcutaneous injections are the standard mode of delivery for insulin therapy; however, this procedure is very often invasive, which hinders patient compliance, particularly for individuals requiring insulin doses four times a day. Furthermore, cases have been reported of sudden hypoglycemia occurrences following multidose insulin injections. Such an invasive and intensive approach motivates the quest for alternative, more user-friendly insulin administration approaches. For example, transdermal delivery has numerous advantages, such as prolonged drug release, low variability in the drug plasma level, and improved patient compliance. In this paper, the authors summarize different approaches used in transdermal insulin delivery, including microneedles, chemical permeation enhancers, sonophoresis, patches, electroporation, iontophoresis, vesicular formulations, microemulsions, nanoparticles, and microdermabrasion. Transdermal systems for insulin delivery are still being widely researched. The conclusions presented in this paper are extracted from the literature, notably, that the transdermal route could effectively and reliably deliver insulin into the circulatory system. Consistent progress in this area will ensure that some of the aforementioned transdermal insulin delivery systems will be introduced in clinical practice and commercially available in the near future.

## 1. Introduction

In the last few decades, diabetes mellitus has emerged universally as an epidemic, and has become the fifth most prominent cause of mortality [1]. 

More than 422 million individuals worldwide have diabetes, according to a WHO report (see: https://www.who.int/health-topics/diabetes#tab=tab_1). This number could increase to 693 million by 2045 if proper actions are not taken [2,3]. In general, there are two classes of diabetes mellitus, i.e., type 1 and type 2. Type 1 is mostly due to a total insulin deficiency, but the causes of type 2 are varying degrees of insulin resistance, impaired insulin secretion, and elevated glucose production. Type 1 may be further subcategorized into type 1A diabetes mellitus, i.e., the autoimmune degradation of β-cells, and type 1B, i.e., idiopathic insulin deficiency [4]. The incidence of diabetes is growing owing to an aging population and improved diagnosis [5,6].

**Figure 1:**
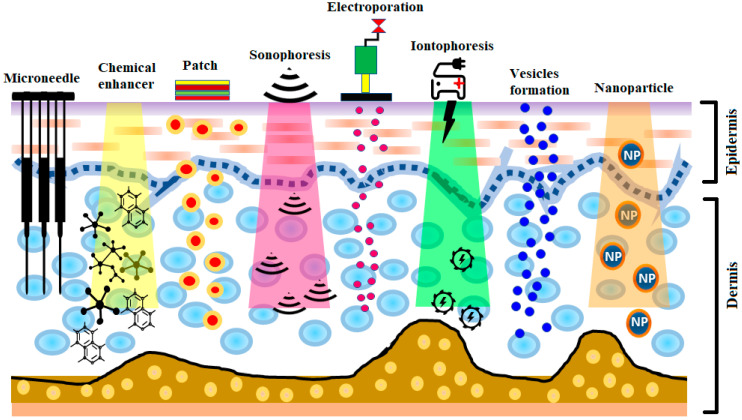
A schematic illustration of various strategies for insulin delivery via the transdermal route.

The development of insulin has been identified as one of the most significant events in the treatment of diabetes. The production of human insulin analogs using recombinant technology was seen as a huge step forward [7]. Insulin therapy has a significant role in treating type 1 diabetes. The subcutaneous route has been the most widely used, as it precludes enzymatic insulin degradation in the digestive tract. Healthy glycemic controls need to be preserved in type 1 diabetes, requiring at least three or maybe more daily insulin shots. Nevertheless, this route comes with the risk of infection and inflammation induced by the use of subcutaneous needles. Later, alternative routes—for instance, pulmonary, nasal, and oral routes—were investigated [8,9]. Pens, jet injectors, sharp needles, supersonic injectors, and infusion pumps have been introduced to minimize pain and enhance adherence to insulin regimens [10,11,12,13]. Still, compared to subcutaneous injections, the insulin absorption from the aforementioned techniques into the blood is quite low, and, consequently, other systems for insulin therapy are required [14,15]. Some noninvasive methods are being explored for insulin delivery [14]. Recently, there has been significant interest in the delivery of drugs via the transdermal route [16]. The transdermal route is an interesting choice for insulin delivery, as this approach would mitigate the pain and infection risk related to subcutaneous injections [17]. Furthermore, the transdermal route ensure patient compliance as well as delivery-controlled insulin release over time [18,19]. Still, the transdermal delivery of drugs is restricted, owing to the low permeability of the stratum corneum [20,21,22,23,24]. In recent years, a number of experimental techniques seeking to improve transdermal insulin delivery have been proposed [18,25,26,27,28,29,30]. In this review, different transdermal insulin delivery techniques and their improvements for diabetes care are highlighted. A schematic illustration of various strategies for insulin delivery via the transdermal route is presented in Figure 1.

## 2. Microneedle

**Figure 2:**
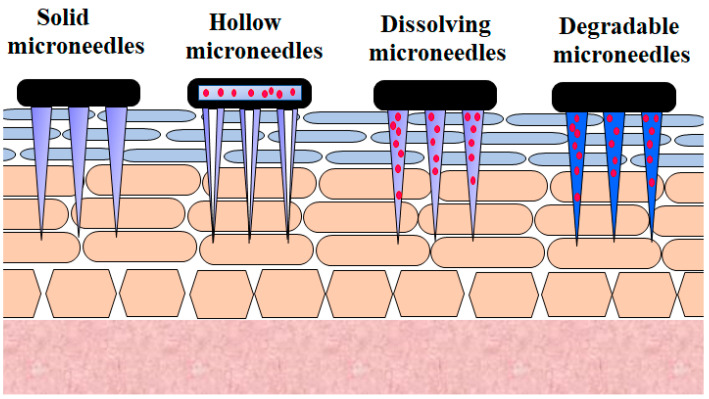
Various microneedles employed for transdermal insulin delivery.

Microneedle technology offers an appealing technique for generating reversible skin microchannels that improves the skin permeability and allows the delivery of a wide variety of biotherapeutics, including insulin [31]. The delivery of microneedles at the site of application causes substantially less anxiety, pain, and tissue harm, owing to their minute size, as opposed to that of the 26-G hypodermic needle [32]. Micrometer-sized needles are sufficiently long to reach the corneum [33,34,35,36]. They are adequately narrow and sharp to cause minimal trauma and decrease the probability of infection [32,37]. This method offers a similar efficiency to standard injections. Besides this, the microneedle approach reduces the inherent problems associated with other invasive techniques [38]. Various microneedles employed for the transdermal delivery of insulin are presented in Figure 2.

Wu et al. prepared an intradermal microdelivery device consisting of a wafer with microneedles (150 μm length) for controlled insulin release. In vitro experiments showed that the level of fluorescein isothiocyanate-labeled insulin penetration after microneedle pretreatment was substantially enhanced and provided numerous upsurges after the donor phase separation. Animal studies established that the antidiabetic pattern of percutaneously administered insulin in rats was similar to that of biphasic insulin injected by the subcutaneous route [39].

The transdermal application of insulin using microneedles made of biodegradable or dissolving polymers has attracted considerable interest [40,41]. The benefit of microneedles made of polymers is that if they break down in the skin, they do not present a safety problem; rather, they simply dissolve or degrade entirely and safely. Furthermore, they are reasonably priced (compared to silicon ones). Other advantages are that drugs can be enclosed inside a microneedle polymer matrix, thereby increasing the drug loading capacity [42]. 

In a recent study, microneedle (dissolving polymeric) patches were developed using sodium carboxymethyl cellulose and gelatin to deliver insulin. The study results indicated that the dissolving microneedles released FITC-insulin rapidly, and then steadily disseminated the insulin into the layers of the skin. This research confirmed that insulin microneedle patches provide adequate relative bioavailability, i.e., similar to that of hypodermic injection [31]. 

Previously, Chen et al. prepared completely insertable microneedles, made from polyvinyl alcohol and poly-c-glutamic acid/polyvinyl pyrrolidone. The authors showed that poly-c-glutamic acid microneedles developed using a guiding structure design permitted the microneedles to be completely inserted and then distributed the insulin efficiently into the tissue. It was observed that innovations in the design of the microneedles yielded a fast, comfortable self-administration strategy for use with insulin or other therapeutic proteins [43]. 

In another study, Ling and Chen produced a patch containing dissolving microneedle made of gelatin and starch that liquefy within five minutes of application, rapidly releasing insulin. The authors reported that the microneedles had adequate mechanical strength to be introduced to a distance of 0.2 mm (in vitro) and 0.2–0.25 mm (in vivo) in porcine and rat skin without causing significant irritation or the sensation of pain. These insulin-loaded, quick-dissolving microneedles were applied to the rats with diabetes for in vivo analyses. Analogous antidiabetic and pharmacokinetic findings were reported in rats treated with these microneedles and a control preparation (insulin s.c. injection). The study concluded that the prepared microneedles had immense promise for the transdermal delivery of insulin [42].

In a previous study, Ito et al. also produced a dissolving microneedle in which 225–300 microneedles laden with insulin were assembled on a chip. Insulin-dissolving microneedles were introduced into the skin by pushing with the hand. The study noted that the depth of penetration of the microneedles was improved from 21 to 63 μm at 0.8 to 2.2 m/s in proportion to the speed of application to the isolated rat skin. Human skin showed comparable findings in terms of the depth of penetration. The developed two-layered dissolving microneedle application framework proved to be helpful for the delivery of transdermal insulin [44].

In 2012, insulin-loaded microneedle arrays made of hyaluronic acid (length: 800 μm; base and tip diameters: 160 and 40 μm, respectively) were prepared and investigated. An animal study using diabetic animals reported a dose-dependent antidiabetic outcome and only minor skin damage caused by the microneedles. The findings showed that the newly designed microneedles could become a popular option for insulin delivery without causing substantial skin injury [45]. 

Zhou et al. investigated the effectiveness of commercially available microneedle rollers having microneedle lengths of 0.25, 0.5 and 1.0 mm for transdermal insulin delivery to diabetic animals. The authors showed that microneedle rollers of 250 and 500 μm were safer and very efficient devices for administering insulin transdermally in rats [46]. 

Still, there are lots of issues associated with microneedles from the perspective of clinical application, and this technique could attain high-efficiency, controllable, and sustained insulin delivery after overcoming such issues. First of all, the safety of the materials should be intensively tested. Second, skin allergies, redness, and irritation are the main issues attributed with the application of microneedle technology. Another drawback associated with this technology is that the microneedle can only be loaded with a small dose of medicine. In addition, if the pores on the skin created by the use of a microneedle do not close after application, there is a risk of infection. Some micromolecules might remain in the microneedles after polymerization, which could compromise the skin tissue or trigger severe reactions. Furthermore, insulin delivery strategies using microneedles should be more deeply examined to achieve the accurate and consistent delivery of insulin, since hypoglycemia could result from an overdose. Also, to begin large-scale production of microneedles and minimize costs, the fabricated approach should be streamlined. Finally, in order to verify that microneedles can effectively function in a physiological setting, additional clinical trials must be considered [47,48,49,50,51].

The various approaches used in the systemic delivery of insulin via the skin are addressed in the article and a summary is tabulated in Table 1.

## 3. Chemical Permeation Enhancer

The term “chemical permeation enhancer” applies to a substance or mixture of substances that enhances the permeability of the skin. Several groups of permeation enhancers have been tested for the delivery of various lipophilic and hydrophilic drugs(s) using both human and animal skins [56,117]. 

Transdermal enhancers (linolenic acid and oleic acid) and microwave techniques have recently been investigated to improve transdermal insulin permeation. The transdermal enhancer, linoleic acid, was the least active in terms of increasing the delivery of transdermal insulin, while the permeability enhancer, oleic acid, was found to be stronger than linolenic acid but failed to provide significant insulin permeation. The best result was found with microwave technology that facilitates insulin absorption and decreases the blood glucose levels in animals [62].

Previously, different chemical permeation enhancers were examined by Yerramsetty et al. to ameliorate skin permeability for the delivery of insulin. Amongst the investigated permeation enhancers, a total of eight, i.e., decanol, menthone, oleic acid, cycloundecanone, cis-4-hexen-1-ol, 2,4,6-collidine, octaldehyde, and 4-octanone, were found to be highly enhancing and nontoxic, five (cis-4-hexen-1-ol, 2,4,6-collidine, cycloundecanone, 4-octanone, and octaldehyde) of which were new discoveries [63].

Rastogi et al. assessed various transdermal enhancers in conjunction with iontophoresis; the findings indicated that the mixtures of oleic acid, 1,8 cineole and sodium deoxycholate in 3:7 ratio of ethanol:propylene glycol contributed to a 45% improvement in insulin permeation in the presence of iontophoresis, as compared to iontophoresis alone. The combinatorial use of iontophoresis with a chemical enhancer contributed to a substantial reduction in the level of glucose in blood for 8 h in rats with diabetes [64]. Rastogi and Singh examined the influence of limonene, linoleic, oleic, palmitic, stearic, palmitoleic, linolenic and iontophoresis on the transdermal insulin delivery via the epidermis of porcine. The authors noted that linolenic acid contributed to better iontophoretic and passive insulin permeability. The research effectively indicated the potential to deliver therapeutic insulin levels through iontophoresis in conjunction with chemical permeation enhancers [65]. 

In 2008 and 2009, Li et al. highlighted the influence of trypsin on the permeation of insulin via skin in rat, without and with trypsin pretreatment. The authors noted that pretreatment with 0.25% trypsin led to a 5.2-fold increase in penetrability [66]. In addition, the FITC-insulin permeation flux was enhanced by 10-fold after pretreatment with trypsin [67]. In a separate study, the addition of permeation enhancers such as dimethyl sulfoxide, azone, or *n*-methyl-2-pyrrolidone into propylene glycol-drug formulations enhanced insulin penetration in an in vitro experiment. At 0.1% and 12.0% concentrations, azone and *n*-methyl-2-pyrrolidone demonstrated optimum efficacy. Dimethyl sulfoxide was reported to have less influence on enhancing transdermal insulin delivery than the other two investigated enhancers [68]. 

In another study, the influence on the transdermal delivery of insulin to rats of terpenes, such as pulegone, menthone, menthol, and cineole in ethanol, without and with iontophoresis, was investigated. With terpene in ethanol, a synergistic enhancement in the insulin flux was noted, while menthone in ethanol showed the highest improvement in flux of insulin among the terpene/ethanol combinations. Meanwhile, neat menthone presented greater insulin enhancement than menthone in ethanol. In contrast to other chemical enhancer pretreatments, iontophoresis had a lower influence on the skin obstacle properties. Terpene in ethanol mediated a synergistic improvement of insulin permeation when combined with iontophoresis [69]. Later, a gel preparation of insulin using poloxamer 407 was prepared by Pillai and Panchagnula. In ex vivo tests, both linoleic acid and menthone demonstrated a synergistic increase in insulin penetration in conjunction with iontophoresis. A decrease in the plasma glucose level of 36–40% was achieved by iontophoresis alone or in conjunction with linoleic acid. The authors noted that more skin discomfort was caused by the chemical enhancers when combined iontophoresis than when each was used alone [70]. 

## 4. Patches

In particular, transdermal patches are an appealing dosage method for the predictable and consistent delivery of insulin into the bloodstream. Insulin patches contribute to patient-friendly, noninvasive and painless delivery of insulin. Even, in the case of hyperinsulinemia, patients can easily remove the patches. Recently, nanoheaters incorporated into insulin patches were shown to effectively release insulin, and demonstrated comparable in vivo activity in mice with respect to s.c. injection of insulin [75].

In 2002, an innovative transdermal lipid-based system (Biphasix-insulin) was produced by King et al. and tested for blood glucose-reducing efficacy in a diabetic rat model induced by streptozotocin. Biphasix-insulin-containing transdermal patches (recombinant human insulin dose 10 mg) were administered to abdominal skin of rats with diabetes for 48 h. A blood glucose level decrease of 43.7%, compared to initial values, was observed. Further, the insulin bioavailability was improved by 21.5%, based on the serum insulin noted from the transdermal Biphasix-insulin patches. It was concluded that the Biphasix device successfully administered insulin via the skin route. These findings support the use of patches containing insulin for human use [29]. In 2003, King et al. reported that insulin in biphasic vesicle-containing transdermal patches had been administered to the abdominal skin of diabetic rats for 73 h, and the levels of blood glucose tested using a glucose meter every 2–10 h. ELISA was used to measure the inguinal lymph node insulin samples. The findings showed that insulin increased in the lymph nodes in a manner that depended on the dose and time. The maximum transdermal lymph node insulin concentrations were reported at 73 h with both 140 IU and 280 IU doses of recombinant insulin. The authors concluded that lymph transport is involved after transdermal insulin delivery of biphasic vesicles [76].

In another study, Mbaye et al. utilized Eudragit RS 100, butylphtalate, and ethyl cellulose to formulate a transdermal insulin system, and found that the continuous release profiles strongly depended on Ethylcellulose/Eudragit [77]. In 2011, Bohannon et al. noted that bolus insulin is easier to obtain, and that it increases quality of life of diabetic subjects [78]. Another study by Qiu et al. described a lyophilized hydrogel patch device for microneedle-mediated insulin delivery. The authors noted that blood glucose was reduced in rats, and action was maintained for longer, compared to subcutaneous injection [79].

In another report, a microfabrication technique was employed to load insulin onta a patch that had 100 dissolving chondroitin sulfate microneedles. By using two or four patches on the (abdominal) skin of dogs, the antidiabetic effects were evaluated, and samples of blood taken, for 6 h. The insulin content per established patch was 1.67 IU. For two patches, a minimum level of glucose plasma was observed at 0.83 h, while for four patch trials, this occurred at 1.37 h. The findings showed that the bioavailability of microneedle insulin was found to be 72.1% for two patches and 72.4% for four patches. Furthermore, researchers noted that insulin was stable at 4 °C for one month in the dissolved microneedles, after which the recovered percentage was found to be 99.2 ± 13.9% [80].

Hadebe et al. studied pectin insulin-containing dermal insulin patches and tested them on diabetic rats. The authors found that the oral glucose test responses of treated rats with transdermal pectin insulin patches displayed reduced blood glucose levels after five weeks, while short-run therapies restored the glycogen levels of both liver and muscle. The produced pectin insulin matrix patch provided regulated insulin release and relieved a variety of diabetic symptoms [19]. In another report, the use of the matrix patch of pectin-insulin was shown to offer protection against the devastating cardiovascular effects associated with the conventional treatment of diabetes [81].

## 5. Sonophoresis

**Figure 3:**
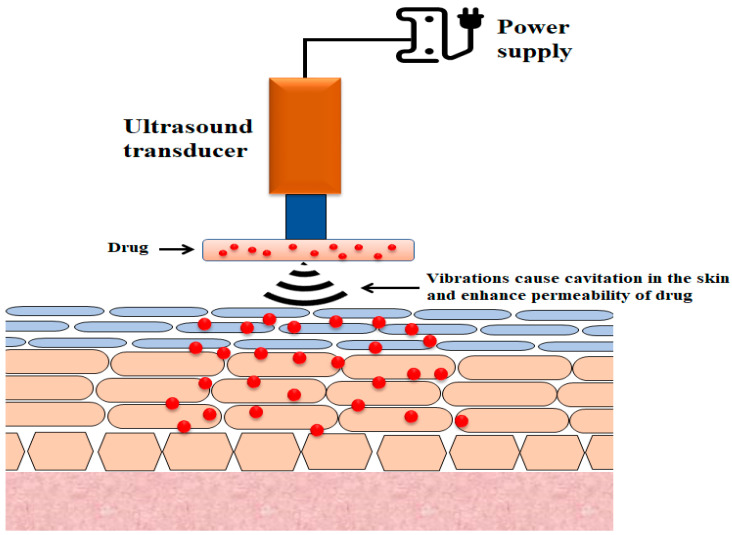
Illustration of the basic design of sonophoretic delivery devices.

The use of ultrasound to enhance transdermal drug transport is referred to as phonophoresis or sonophoresis (Figure 3) [118]. It was observed that the increase in insulin delivery by the skin route that resulted from the use of ultrasound waves (low-frequency 20–100 kHz) could be due to the disruption of stratum corneum layers [119,120]. While sonophoresis has received considerable attention from researchers, its mechanisms are not fully understood, although several probable mechanisms have been suggested, the most plausible of which is cavitation [121,122]. 

The air ultrasonic ceramic transducer for insulin transdermal delivery was developed by Jabbari et al. In their study, rats were divided into four groups: Group 1 was a control group; Group 2 received a subcutaneous injection of insulin (0.25 U/Kg); Topical and ultrasonic transdermal insulin were administered to groups 3 and 4 respectively. The investigators indicated that the application of ultrasound technique ameliorated insulin delivery, and that the diabetic glucose level of rats was reduced to normal values [86]. In another study, the involvement of cavitation in the delivery of transdermal insulin was explored, and substantial improvement in insulin penetration (40%) was reported [87]. In past research, the physiological reaction to transdermal insulin delivery mediated by ultrasound was shown to correlate with that of insulin administered subcutaneously. In this study, blood glucose levels declined by 190 ± 96 mg/dL 1.5 h after insulin administration (subcutaneous injection 0.25 U/kg dose), whereas blood glucose decreased by 263 ± 40 mg/dL at 1.5 h with insulin delivered with ultrasound [88].

## 6. Electroporation

In this process, short, high-voltage electric field signals generate transient aqueous paths in the stratum corneum [123,124,125,126]. Researchers showed that electroporative pulses could be used in diabetic rabbits to regulate blood sugar by improving insulin transportation through the skin. It was highlighted that the increment in insulin dose and electroporative pulses, and decrease in the field strength of electroporation, contributed to a dramatic reduction in the blood sugar levels [90]. In the original report, investigators used a combination of electroporation and iontophoresis to study insulin permeation in rats. The investigators found that the combination of these techniques led to an increase in insulin plasma levels in comparison to those reported following electroporation [91]. In another study, an in vivo potency of the electroporation of insulin as a solution, insulin solution (s.c.), nanoparticle (i.v.) and nanoparticles (electroporation) was discussed. These findings indicated that polymeric nanosystem electroporation was an attractive substitute to injectable administration [127]. Other research indicated that, compared to electroporation alone, electroosmosis combined with electroporation in the presence of 1,2-dimyristoylphophatidylserine (a saturated anionic lipid) contributed a substantially higher transport rate of insulin [92]. 

## 7. Iontophoresis

One possible method for the improvement of drug delivery is transdermal iontophoresis [128,129]. Hao et al. stated that transdermal insulin delivery could potentially be achieved by combining iontophoresis and some enhancers [96]. Elsewhere, transdermal insulin delivery through porcine epidermis was observed by combining the use of iontophoresis with different chemical enhancers [64,65]. In 2002, researchers demonstrated the application of simultaneous techniques, such as iontophoresis + electroporation, for insulin permeation augmentation through human cadaver skin ex vivo [97]. Transdermal insulin delivery using the technique of iontophoresis was demonstrated in [26,69,130,131,132,133]. In a prior study, pretreatment (wiping) of skin using alcohol before iontophoresis was said to produce an impressive increment in insulin transdermal transport [98].

Kajimoto et al. indicated that a progressive drop in the levels of blood glucose in rats with diabetes occurred when employing insulin- liposome and iontophoresis combinations [99]. In other research, the authors verified that the permeation of insulin-loaded, positively-charged nanovesicles—applied via iontophoresis to skin with microchannels created by a microneedle—was 713.3 times greater, compared to the use of nanovesicles. Animal experiments showed that the level of blood glucose in rats with diabetes dropped considerably (comparable to an s.c. injection of insulin) at 4 h and 6 h following the combined application of positive nanovesicles driven by iontophoresis and microneedles [100]. 

## 8. Vesicular Formulations

Liposomes are widely-studied nano-sized lipid vesicles that could be beneficial in the delivery of drugs via the dermal or transdermal routes. Liposomes, as drug carriers, offer many advantages that are reported elsewhere [134,135,136].

Techniques such as using combinations of two or more enhancers or the liposomal formula of insulin were investigated by Ogiso et al. The highest blood sugar lowering action that continued up to 10 h was exhibited by a transdermal system comprising liposomes insulin, d-limonene, and taurocholate. A high hypoglycemia effect was also achieved with a blend of *n*-octyl-beta-d-thioglucoside, cineol, and deoxycholate, or d-limonene and *n*-octyl-beta-d-thioglucoside. The authors clearly showed that the absorption of insulin in the stratum corneum of rat skin could be achieved under certain circumstances [103].

Transferosomes are highly elastic vesicles that are comprised of phospholipids and edge activators [137,138]. They can permeate the skin and are an efficient means by which to deliver entrapped drugs when applied in nonoccluded circumstances [139]. The highly flexible vesicles, named “transferosomes”, were shown to be good drug carriers if loaded with insulin and applied in acceptable quantities [28,104].

The rotary evaporation sonication technique was used to prepare a transdermal transferosome insulin gel. The results showed that the optimized formulation entrapment efficiency of insulin was 78%, and a cumulative release of insulin of 83.11% was observed. In vivo tests clearly showed that a better effect on glucose reduction was achieved by insulin transferosome gels compared to a control gel. The authors concluded that their study showed that the prepared transferosomal formulation could be used as a potential carrier for insulin and other protein deliveries [105]. In an earlier study, optimized insulin-containing transferosome gels were also produced on the basis of a factorial design that showed good permeation flux, i.e., 13.50 ± 0.22 μg/cm^2^/h, across ear skin of porcine. An increased in permeation flux of 17.60 ± 0.03 μg/cm^2^/h was obtained by further applying the technique of insulin transferosomes with iontophoresis. The animal study revealed that the best transferosome gels exhibited sustained hypoglycemic effects over 24 h in diabetic rats [106]. 

## 9. Microemulsion

Microemulsions typically droplets of less than 100 nm in size, and are thermodynamically stable clear liquids [140,141,142]. Microemulsions have been extensively studied and have gained considerable interest as vehicles of transdermal administration [143,144,145,146,147,148].

In 2013, insulin emulgel was prepared using emu oil (composed of fatty acids obtained from emu, a bird, *Dromaius Novae-Hollandiae*, native to Australia) as a permeation enhancer. The biological activity of emulgel alone and in combination with iontophoresis was tested using albino rabbits. The authors claimed that the optimized formulation [F4: emu oil (7.5% *w*/*w*) and polysorbate 80 (5.0% *w*/*w*)] showed a maximum insulin permeation flux of 4.88 μg/cm^2^/h through rat skin. A pharmacodynamic study indicated that the blood glucose level decreased from an initial value of 250 mg/dL to 185 mg/dL and the initial value to 125 mg/dL in 2 h in the group treated with insulin emulgel alone and insulin emulgel + iontophoresis respectively [110]. 

Transdermal microemulsions containing insulin were formulated with 10% oleic acid, 50% surfactant phase, and 2% DMSO, providing a maximum flux of 4.93 μg/cm^2^/h across goat skin. The authors concluded that there was considerable potential to use microemulsions for insulin delivery via the skin route [111].

## 10. Nanoparticles

Earlier, the feasibility of the use of transdermal insulin nanoparticles by means of a supercritical antisolvent micronisation procedure was investigated. The authors indicated that the supercritical antisolvent procedure provided uniform spherical insulin nanoparticles of particle sizes 68.2 ± 10.8 nm. The research indicated that the supercritical antisolvent process did not cause insulin degradation. An in vitro evaluation revealed that the insulin nanoparticles followed Fick’s first diffusion law, and displayed a good permeation rate. The authors found that the prepared nanoparticles containing insulin may have promising possibilities for the transdermal delivery of diabetes chemotherapy [115].

## 11. Microdermabrasion

Microdermabrasion has been previously used to minimize the presence of wrinkles, scars, and fine lines [149,150,151]. Previously, this approach was adopted as a tool to mitigate the hindering nature of the stratum corneum [152,153,154,155]. 

The application of microdermabrasion to improve skin insulin permeability was investigated by Andrews et al., who highlighted that microdermabrasion could improve the permeability of the skin to insulin at levels that are adequate to stabilize the range of blood glucose in rats with diabetes [116]. 

## 12. Conclusions

Chronic diseases such as diabetes are among the most prominent causes of mortality, morbidity, and high health-care costs. For individuals with diabetes, regular shots of insulin are required to maintain normal blood glucose levels. Other techniques for insulin delivery possess some disadvantages. Hence, there is a vital need to find new approaches for insulin delivery. Transdermal drug delivery presents exciting possibilities, as it eliminates the pain and risk of infection associated with subcutaneous insulin injections, ensures patient compliance, and provides a controlled release of insulin. It was observed from the literature that the different transdermal techniques reported in this article have been widely and successfully used for the delivery of insulin. Encouraging developments are occurring which will lead to the more successful and safe delivery of insulin via the transdermal route. 

## Figures and Tables

**Table 1:** Various approaches used in the systemic delivery of insulin via the skin.

Technique	Advantages/Disadvantages	Transdermal Research	Reference
**Microneedle**	*Advantages*: Possible to deliver large molecules, noninvasive delivery, more efficiently regulates the range of drug delivery, and rapid recovery at the application site compared to a hypodermic needle injection [52,53,54,55].	Wu et al. prepared an intradermal microdelivery device consist of a wafer with microneedles.	[39]
		Dissolving polymeric microneedle patches were developed using sodium carboxymethyl cellulose and gelatin.	[31]
		Chen et al. introduced completely insertable microneedle.	[43]
		Patch contained dissolving microneedle, comprising of gelatin and starch that could liquefy quickly in five minutes.	[42]
	*Disadvantages*: Lower dose precision than hypodermic needles, frequent application might trigger tissue injury, tip of the microneedle may split and stay inside the layer, and less than 1 mg of medicine may be administered by bolus [55].	Insulin dissolving microneedles (assembled on a chip) were introduced into the skin by pushing with the hand.	[44]
		Insulin-loaded microneedle arrays made of hyaluronic acid was prepared.	[45]
		Zhou et al. indicated that microneedle rollers of 250 and 500 μm are safer and more efficient devices for administering insulin through the transdermal route in rats.	[46]
**Chemical** **Permeation** **Enhancer**	*Advantages*: Design flexibility, easy implementation, and inexpensive [56,57,58]. *Disadvantages*: Lower potency, and not all are adequate for the delivery of macromolecules and could irritate the skin [59,60,61].	Linolenic acid and oleic acid (enhancers) and the microwave technique were investigated to improve transdermal insulin permeation.	[62]
		Enhancers such as, menthone, decanol, oleic acid, cycloundecanone, cis-4-hexen-1-ol, 2, 4, 6-collidine, octaldehyde, 4-octanone, were found highly enhancing and nontoxic.	[63]
		The mixtures of 1,8 cineole, oleic acid, and sodium deoxycholate in propylene glycol: ethanol (7:3) lead to in a 45% improvement in insulin permeation in the presence of iontophoresis.	[64]
		Enhancers for instance, limonene, oleic, linolenic, palmitic, palmitoleic, linoleic, stearic, and iontophoresis were investigated.	[65]
		Consequence of trypsin as biochemical enhancer was investigated for the transdermal delivery of insulin.	[66,67]
		Permeation enhancers such as *n*-methyl-2-pyrrolidone or dimethyl sulfoxide, azone into the propylene glycol-drug formulations enhanced the in vitro permeation of insulin.	[68]
		Studied terpenes such as cineole, pulegone, menthone, and menthol, in ethanol without and with iontophoresis.	[69]
		Both enhancers such as linoleic acid and menthone demonstrated a synergistic increase in insulin penetration in conjunction with iontophoresis.	[70]
**Patches**	*Advantages*: Convenient to use, patient satisfaction, reduced amounts of medicine may be needed, lower incidences of medication error, and easy withdrawal of the patch in case of any side effects [71,72,73]. *Disadvantages*: Slow time to peak plasma levels, skin irritation; in order to be successful, patches need effective adhesion to the skin; factors such as hairs, oil and sweat on the skin hamper the adhesion of the patch and that could lead to changes in the insulin absorption [73,74].	Incorporation of nano-heaters into insulin transdermal patches allows efficient insulin release.	[75]
		Transdermal lipid-based system (Biphasix-insulin) was produced by King et al; persistent fall in blood glucose level in rats with diabetes was noted.	[29]
		King et al introduced insulin in biphasic vesicles-containing transdermal patches.	[76]
		Mbaye et al. developed a transdermal system of insulin using ethyl cellulose, Eudragit RS 100, and butylphtalate.	[77]
		Bohannon et al. compared the effectiveness, safety, device satisfaction, and quality of life of people with diabetes with an insulin bolus patch.	[78]
		Lyophilized hydrogel patch device for microneedle-mediated insulin delivery formulated.	[79]
		Microfabrication technique was employed for loaded insulin on a patch that had 100 dissolving chondroitin sulfate microneedles.	[80]
		Hadebe et al. studied pectin insulin containing dermal insulin patches and tested in diabetic rats.	[19]
		Pectin-insulin matrix patch ameliorated the diabetes indications in diabetic rats.	[81]
**Sonophoresis**	*Advantages*: Permits the regulation of permeation rates, enables an immediate end of the drug delivery, less irritant, low infection risk, and less painful than an injection [82,83,84]. *Disadvantages*: Complicated, slight tingly and burning sensation, and discomfort [85].	Jabbari et al. prepared an air ultrasonic ceramic transducer for transdermal insulin delivery.	[86]
		The involvement of cavitation in the delivery of transdermal insulin was explored.	[87]
		Park et al. reported that the fall in the blood glucose levels was considerably higher in the group treated with ultrasound than treated with subcutaneous injection.	[88]
**Electroporation**	*Advantages*: Permits control of the permeation rates, delivery can be ceased immediately, less irritants, and less painful [89]. *Disadvantages*: Cell damage, time-consuming, nonspecific [89].	Investigators demonstrated that the electroporative pulses could be used in diabetic rabbits to regulate the blood sugar by improving insulin transportation through the skin of the rabbit.	[90]
		Demonstrated the beneficial consequences of electroporation and iontophoresis on human insulin permeation in rats.	[91]
		The in vivo potency of the electroporation of insulin as a solution, insulin solution (s.c.), nanoparticle (i.v.) and nanoparticles (electroporation) was investigated.	[92]
**Iontophoresis**	*Advantages*: used for unionized and ionized and high molecular weight molecules, delivery can be ceased immediately, and improved control over drug delivery [93,94,95]. *Disadvantages*: time-consuming and, might be harmful for budding hair [93,94,95].	Indicated that the insulin delivery by skin route could be achieved by the combining of iontophoresis and some enhancers.	[96]
		Transdermal insulin delivery through the porcine epidermis was demonstrated by combining iontophoresis with different chemical enhancers.	[64,65]
		Simultaneous technique, such as iontophoresis + electroporation, were investigated for increasing insulin permeation through human cadaver skin.	[97]
		Demonstrated that pretreatment (wiping) of skin with ethanol before iontophoresis produced an impressive increment in the transdermal transport of monomeric insulins.	[98]
		Kajimoto et al. used charged liposomes and optimized iontophoretic parameters for transdermal insulin delivery.	[99]
		Iontophoresis-driven insulin from nanovesicles via a microchannel induced by microneedles in the skin, boosts the transdermal delivery of insulin.	[100]
**Vesicular forMulations**	*Advantages*: versatile system could entrap the miscellanea of medicine, biocompatible, biodegradable, and provide sustained drug release [21,101,102]. *Disadvantages*: leakage, lower stables, the purity of natural phospholipids is also another obstacle, and expensiveness [21,101,102].	Liposomal insulin combined with d-limonene and taurocholate showed the maximum hypoglycemic effect.	[103]
		Demonstrated the use of ultra-deformable carriers for transdermal insulin delivery.	[104]
		Investigator demonstrated that transferosomes could be used as potential carrier for insulin.	[105]
		Factorial design-based-optimized insulin-transferosomes gel has shown 13.50 ± 0.22 μg/cm^2^/h transdermal flux across porcine ear skin.	[106]
**Microemulsion**	*Advantages*: thermodynamic stability, the increased solubility and stability of drugs, versatile carrier, and economical scale-up [107,108,109]. *Disadvantages*: leakage, phase inversion, and needs the development of complex systems that could be time consuming.	Insulin emulgel was prepared using emu oil.	[110]
		Insulin-loaded microemulsion composed of oleic acid (10%), aqueous phase (38%), and surfactant phase (50%) with dimethyl sulfoxide (2%) was prepared and evaluated.	[111]
**Nanoparticles**	*Advantages*: sustained drug release, due to their nano-size particles permeate efficiently across the skin, and low-irritancy [112,113,114]. *Disadvantages*: expensive and the formulation requires special and expensive techniques [112,113,114].	Prepared transdermal insulin nanoparticles by means of a supercritical anti-solvent micronization procedure.	[115]
**Microdermabrasion**	*Advantage*: efficiently increases the permeability of the drug. *Disadvantages*: expensive and, skin irritation.	Andrews et al investigated the microdermabrasion technique to improve the skin insulin permeability.	[116]

## Data Availability

Not applicable.
